# Dependence of *Mycoplasma bovis* on a novel nucleoside transporter for survival in association with host cells

**DOI:** 10.1128/aem.01298-25

**Published:** 2025-12-12

**Authors:** Shijie Geng, Chintha K. Premachandre, Shukriti Sharma, David P. De Souza, Sheik Nadeem Elahee Doomun, Jordi Hondrogiannis, Anna Kanci Condello, Glenn F. Browning, Sara M. Klose, Kelly A. Tivendale, Nadeeka K. Wawegama

**Affiliations:** 1 Asia-Pacific Centre for Animal Health, Melbourne Veterinary School, Faculty of Science, The University of Melbourne 2281, Parkville, Victoria, Australia; 2 Metabolomics Australia, Bio21 Institute of Molecular Science and Biotechnology, The University of Melbourne 2281, Parkville, Victoria, Australia; Washington University in St. Louis, St. Louis, Missouri, USA

**Keywords:** *Mycoplasma bovis*, transporter, metabolomics, metabolism

## Abstract

Transport systems play a vital role in metabolism in mycoplasmas by facilitating the uptake of essential nutrients from host cells, on which mycoplasmas are highly dependent. Co-culture of *Mycoplasma bovis* with Madin-Darby bovine kidney cells has shown that the gene MBOVPG45_0748 is essential for the survival of *M. bovis* in association with host cells. To characterize the function of this protein, intracellular and extracellular metabolomic profiles of a mutant strain with a transposon inserted into the MBOVPG45_0748 gene (∆MBOVPG45_0748) were compared to those of the parent strain, PG45. The ∆MBOVPG45_0748 mutant contained lower levels of metabolites involved in purine, pyrimidine, and glycerol metabolism and elevated levels of intermediates in the pentose phosphate and glycolysis pathways. Depletion of nucleosides from the culture supernatant occurred at a significantly reduced rate in the mutant, suggesting a role of the product of MBOVPG45_0748 in nucleoside uptake. This was further supported by isotope labeling studies with ^13^C_5_-thymidine, which confirmed that nucleoside uptake and interconversion were disrupted in ∆MBOVPG45_0748 and suggested a compensatory strategy for the maintenance of nucleic acid biosynthesis in the mutant. The poorer survival of ∆MBOVPG45_0748 in medium with DNA supplementation showed that the MBOVPG45_0748 protein was involved in the efficient import of nucleosides derived from degradation of extracellular DNA. Overall, these findings suggest that the MBOVPG45_0748 protein is a nucleoside transporter involved in the uptake of purines and pyrimidines and maintenance of their intracellular balance, which is essential for the survival of *M. bovis* in association with host cells.

**IMPORTANCE:**

*Mycoplasma bovis* causes pneumonia, mastitis, arthritis, keratoconjunctivitis, and reproductive tract disease in cattle, compromising animal welfare and imposing economic losses on farmers. Development of effective control strategies against *M. bovis* requires a better understanding of the host-microbe interactions involved in the pathogenesis of the diseases it causes. Mycoplasmas are dependent on transport systems to acquire nutrients from host cells. Thus, these systems play an important role in their survival and virulence. We used a combination of metabolomic techniques to establish that a transporter gene that is essential for the survival of *M. bovis* in association with host cells plays a role in nucleoside uptake. These results highlight the importance of purine and pyrimidine metabolism in the interactions between *M. bovis* and host cells.

## INTRODUCTION

*Mycoplasma bovis* is an important pathogen of cattle, causing mastitis, pneumonia, middle ear infections, and arthritis (1). The disease and the reduced productivity it causes in dairy and feedlot cattle result in animal welfare issues and substantial economic loss (2). Because infections with *M. bovis* are typically chronic, treatment often involves prolonged antimicrobial therapy, which may increase the likelihood of selection for antimicrobial resistance in *M. bovis* (3, 4). Therefore, preventative measures, such as biosecurity and vaccination, remain the most effective strategies for controlling disease caused by *M. bovis* (5). However, the efficacy of commercially available *M. bovis* vaccines and vaccine candidates has not been established (6, 7), and it is likely that more effective vaccines need to be developed. Achieving this requires a better understanding of the pathogenesis of *M. bovis*, including characterization of the functions of genes implicated in host-pathogen interactions, including those with metabolic roles.

Because they lack a number of the biosynthetic and metabolic pathways found in other bacteria, mycoplasmas are heavily reliant on transport systems to acquire essential nutrients from their host’s cells (8), including glycerol (9), sugars (10), purines and pyrimidines (11, 12), and these transporters play a vital role in their metabolism and survival. Transport systems can also play a role in virulence in mycoplasmas, exemplified by the role of glycerol transport in hydrogen peroxide production (13, 14). While genomic analyses have identified multiple predicted transporters in *M. bovis* (15), the molecules they transport and the roles they play in interactions with host cells are yet to be fully elucidated. Gene annotations are often based on sequence similarity, and the predicted functions for these putative transport systems are based on characterized transporters in the database. This may result in limited or even misleading descriptions of the roles of these transport systems. Metabolomics has been used to assist in functional characterization of genes in mycoplasmas involved in metabolism and nutrient transport (9, 16). For instance, in *M. bovis*, metabolomic analyses revealed that a putative transporter initially annotated as an amino acid transporter is, in fact, a nucleotide sugar transporter and that one initially annotated as a dicarboxylate/amino acid symporter is a biopterin/folate transporter (16).

Co-culture assays examining the survival and replication of transposon mutants of *M. bovis* PG45 in association with Madin-Darby bovine kidney (MDBK) cells have shown that the MBOVPG45_0748 gene is essential for the survival and proliferation of *M. bovis* in association with host cells (17). This gene is located in a putative glycerol ATP-binding cassette (ABC) transporter operon. The function of the adjacent gene in this operon, MBOVPG45_0749, was examined previously using ^13^C-glycerol in stable isotope labeling studies (16), but no significant difference in glycerol uptake was detected between the parent strain PG45 and the ∆MBOVPG45_0749 transposon mutant (16), suggesting that the function predicted by bioinformatic analysis may not be correct. In this study, the function of the MBOVPG45_0748 gene was investigated using a combination of intracellular and extracellular metabolomic profiling to identify the potential target nutrients of this transporter.

## MATERIALS AND METHODS

### Bioinformatic analysis

Operon analysis and prediction of gene functions were performed using the BioCyc database (18). To assist functional prediction, conserved domains and motifs in the predicted protein sequences were identified using the Conserved Domains Database in the National Center for Biotechnology Information (19) and Kyoto Encyclopedia of Genes and Genomes (KEGG) database (https://www.genome.jp/kegg/genes.html). Homologs were also predicted using the web-based Protein Homolog/analogY Recognition Engine (v.2.0) (Phyre^2^) server (20), and an online topology prediction server, DeepTMHMM (https://dtu.biolib.com/DeepTMHMM), was used to identify transmembrane regions in the protein sequence.

### *M. bovis* strains and culture

Frozen stocks of *M. bovis* strain PG45 were inoculated at a 1:10 dilution into *M. bovis* culture medium (0.6% heart infusion broth, 1% protease peptone no. 3, 0.5% NaCl, 3.7% yeast extract, 10% inactivated swine serum, 0.0064% phenol red, and 300 µg penicillin/mL, pH adjusted to 7.8). The cultures were incubated for 24 h at 37℃, yielding cultures with a titer of 9.77 × 10^8^ color-changing units (CCU)/mL. Titrations to determine concentrations of viable mycoplasmas were performed using the most probable number (MPN) method, as previously described (16). Briefly, 10-fold serial dilutions of cultures were prepared in 96-well plates in *M. bovis* culture medium. The plates were then incubated at 37℃ for up to 14 days, and the MPN of mycoplasmas in the original culture was calculated based on the color change observed in the wells of the plates.

The *M. bovis* mutant strain, hereafter referred to as ∆MBOVPG45_0748, was generated previously by transposon mutagenesis (15). Its genome contains a transposon Tn*4001* insertion in the MBOVPG45_0748 locus between nucleotides 873,250 and 873,251. Gentamicin sulfate (50 µg/mL) was added to *M. bovis* culture medium when culturing ∆MBOVPG45_0748 to maintain selection for carriage of the transposon, and the same culture conditions as those used for the parent strain were used to obtain stocks of ∆MBOVPG45_0748 with a titer of 6.17 × 10^8^ CCU/mL (15). PCR amplification and Sanger sequencing were used to confirm the presence of the transposon in the mutant, as described previously (16). Briefly, a primer complementary to MBOVPG45_0748 (GCTTTTGGCTGGACACTGTTA) was paired with the IR inverse primer complementary to the transposon (TGGCCTTTTTACTTTTACACAAT) to amplify the PCR product using Platinum Taq DNA polymerase (Invitrogen) according to the manufacturer’s instructions. The PCR products were subjected to agarose gel electrophoresis and Sanger sequencing. The sequences were aligned with the genome sequence of *M. bovis* PG45 (GenBank accession number CP002188) to confirm the transposon insertion in MBOVPG45_0748.

### Growth curves of *M. bovis* PG45 and ∆MBOVPG45_0748 strains in axenic medium

To ensure that the metabolic activity was consistent and the biomass was optimal for further metabolomic analyses, the mid-logarithmic phases of growth of PG45 and ∆MBOVPG45_0748 were identified by determining their growth curves (Fig. S1). Stocks of both strains were diluted 1:40 in *M. bovis* growth medium and cultured at 37°C. Titers of viable organisms were determined immediately after inoculation, and then every 2 h from 10 to 18 h after inoculation. The mid-logarithmic phase was reached at 14 h after inoculation for PG45 and at 10 h after inoculation for ∆MBOVPG45_0748.

### Steady-state metabolomic analysis using gas chromatography-mass spectrometry

Biological replicates of PG45 and ∆MBOVPG45_0748 were diluted 1:40 in 10 mL of *M. bovis* growth medium and incubated at 37°C until they reached the mid-logarithmic phase of growth. A 500 µL sample was removed for titration, and then the cultures were quenched by infusing them with 28.5 mL of ice-cold 1× PBS and incubating the infused culture in an ice-water slurry for 5 min. To obtain intracellular metabolites, mycoplasma cells were collected by centrifugation (20,000 × *g*, 2°C, 20 min) and then washed twice with ice-cold 1× PBS (17,000 × *g*, 2°C, 5 min). The cell pellets were resuspended in 50 µL of pure chloroform, then 200 µL of a 3:1 mixture of methanol/H_2_O containing the internal standards (^13^C_6_-sorbitol and ^13^C_5_^15^N-valine) was added. The mixtures were agitated at 4°C for 10 min and then centrifuged at 16,048 × *g* at 4°C for 10 min. A 100 µL volume of each sample was evaporated at 30°C to complete dryness using a Christ RVC 2-33 CDplus rotary vacuum concentrator. Methods used for metabolite derivatization and gas chromatography (GC)-mass spectrometry (MS) analysis for mycoplasma cells were adapted from a previous study (21). In brief, dried samples were derivatized by mixing them with 25 µL of methoxyamine hydrochloride and then incubating the samples, with shaking, at 37°C for 2 h, then adding 25 µL of *N*, *O*-bis(trimethylsilyl) trifluoracetamide with trimethylchlorosilane and incubating them at 37°C for 1 h. A 1 µL volume of each derivatized sample was injected onto an Agilent DB-5 column in a 2030 Shimadzu gas chromatograph coupled with a TQ8050NX triple quadrupole mass spectrometer (Shimadzu, Japan). Metabolites were identified by comparing the retention time and molecular mass to the endogenous metabolites in the Shimadzu Smart Metabolite Database, and data matrices with the relative peak areas for each metabolite were generated for further data processing.

### Metabolomic footprinting using GC-MS

Metabolomic footprinting was performed as described previously, with minor changes (21). In brief, mycoplasma cells were harvested by centrifugation (18,700 × *g*, 20 min, 2°C) from 100 mL of mid-logarithmic phase cultures of PG45 and ∆MBOVPG45_0748. The cells were then resuspended in 1 mL of fresh *M. bovis* growth medium, and the culture was incubated at 37°C for up to 60 min. A 30 µL sample of the culture was collected at 0, 15, 30, and 60 min. The samples were immediately quenched in a dry ice-ethanol bath, then centrifuged at 17,000 × *g*, 0°C, for 10 min to obtain the culture supernatant for the metabolomic analysis of the extracellular components in the medium. Three biological replicates were generated and analyzed for each *M. bovis* strain. Extraction of metabolites from the supernatant and GC-MS detection were performed as described above.

### Stable isotope labeling analysis using ^13^C_5_-thymidine

Stable isotope labeling was performed based on the method described previously (21). In brief, three biological replicates of PG45 and ∆MBOVPG45_0748 were cultured in *M. bovis* growth medium with 32 µM ^13^C_5_-thymidine until mid-logarithmic phase. Metabolic quenching and extraction were performed as described above for the GC-MS platform. For liquid chromatography (LC)-MS detection, mycoplasma cell pellets were resuspended in 250 µL of chilled chloroform:methanol:Milli-Q water (1:3:1) containing 2 µM ^13^C_5_^15^N-valine and 2 µM leucine-d_3_ as internal standards. Samples were vortexed and incubated at 4°C for 10 min with continuous agitation using a Thermomixer C (Eppendorf), then centrifuged at 16,048 × *g*, 4°C, for 10 min. A 225 µL sample of the supernatant was transferred into high-performance liquid chromatography inserts and evaporated under nitrogen using the MICROVAP Nitrogen Evaporation System. The dried extracts were resuspended in 75 µL of acetonitrile:water (4:1), and the metabolites were detected on a Vanquish Horizon UHPLC system (Thermo Fisher Scientific) coupled to an Orbitrap ID-X Tribrid mass spectrometer (Thermo Fisher Scientific). Metabolites were identified using TraceFinder (Thermo Fisher Scientific) and El-Maven (https://www.elucidata.io/el-maven) by matching them using accurate mass and retention time measurements to the 550 authentic standards in the Metabolomics Australia in‐house library. Metabolites with a peak area of the labeled isotopologue pool greater than 10,000 in the generated data matrix were identified as labeled to avoid background noise effects.

### Determining the ability of *M. bovis* to utilize extracellular DNA

Strains PG45 and ∆MBOVPG45_0748 were diluted 1:40 in *M. bovis* growth medium and cultured at 37°C for 18 h. Cells from a 1 mL sample of the cultures were harvested by centrifugation (10,000 × *g*, 5 min) and washed once with 1× PBS. The cells were then resuspended to a final concentration of 10^4^ CCU/mL in MDBK cell culture maintenance medium composed of Advanced Dulbecco’s Modified Eagle Medium (DMEM)/F12 (Gibco)-based cell maintenance medium containing 10 mM N-2-hydroxyethylpiperazine-N-2-ethane sulfonic acid, 0.5% fetal bovine serum, and 100 µg penicillin/mL or in MDBK cell culture maintenance medium supplemented with DNA (0.002% herring sperm DNA). Samples were incubated in 48-well cell culture plates, with some wells containing cell culture maintenance medium alone as the negative controls and others containing suspensions of PG45 or ∆MBOVPG45_0748 in each of the different cell culture maintenance media. Three biological replicates were analyzed. The cell culture plates were incubated at 37°C in 5% CO_2_; samples were collected at 0, 24, 48, and 72 h after inoculation; and the titers of viable *M. bovis* in each sample were determined.

### Statistical analysis

Statistical analysis of the metabolomic data matrix generated by GC-MS was performed using MetaboAnalyst (https://www.metaboanalyst.ca). In order to identify changes in the relative abundance of metabolites between strains, the fold change (FC) of each metabolite in the steady-state metabolomic analysis was calculated by dividing the average median-normalized peak area in ∆MBOVPG45_0748 by that of the parent strain. To determine metabolite changes over time in the metabolomic footprinting analysis, the FC of each metabolite was calculated by dividing the median-normalized peak area at 15, 30, and 60 min by that at 0 min. GraphPad Prism (v.9.4.1) was used to perform unpaired *t*-tests to determine the significance of differences between the two strains. To assess the effect of inclusion of DNA in the medium on the growth of the mutant, two-way analysis of variance and Dunnett’s multiple comparison tests were used to compare differences in titers of viable mycoplasmas between the strains at different timepoints and in different types of media. A *P* value of <0.05 was considered significant.

## RESULTS

### Bioinformatic analysis indicated that MBOVPG45_0748 encodes a permease protein in a putative glycerol transporter operon

**Fig 1:**
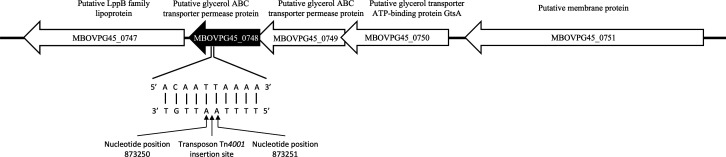
Schematic diagram of the transporter operon containing MBOVPG45_0748, showing the location of the transposon insertion. Putative gene functions were obtained from the BioCyc database.

Operon analysis in the BioCyc database showed that MBOVPG45_0748, which encodes a putative permease protein, lay within a putative glycerol ABC transporter operon, with three genes located upstream, coding for a putative permease protein (MBOVPG45_0749), a putative ATP binding protein GtsA (MBOVPG45_0750), and a putative membrane protein (MBOVPG45_0751), and one gene located downstream, coding for a putative LppB family lipoprotein (MBOVPG45_0747) (Fig. 1). The KEGG database predicted that MBOVPG45_0748 contained a binding-protein-dependent transport system inner membrane component (BPD_transp_1) motif located between amino acids 95 and 261. Conserved domain analysis predicted that it contains a UgpE superfamily domain of an ABC-type glycerol-3-phosphate transport system. Analysis using the Phyre^2^ server found that 97% of the amino acid sequence of MBOVPG45_0748 (263 residues) could be modeled with 100% confidence to the crystal structure of a *Sphingomonas* sp. SKA58 ABC transporter, while analysis of the predicted transmembrane topology of MBOVPG45_0748 using DeepTMHMM detected six transmembrane structures (Fig. S2).

### Disruption of MBOVPG45_0748 alters the intracellular metabolome of *M. bovis*

GC-MS detected a total of 117 intracellular metabolites in both the parent strain PG45 and the ∆MBOVPG45_0748 mutant. Principal component analysis indicated that the parent strain and the mutant aggregated into separate clusters, with the first and second principal components accounting for 35.5% and 20.7% of the total variance, respectively (Fig. S3). A total of 48 metabolites differed in abundance between ∆MBOVPG45_0748 and the parent strain, with 16 higher in abundance (FC > 1.3, *P* < 0.05) and 32 lower in abundance (FC < 0.77, *P* < 0.05) (Table S1).

**Fig 2:**
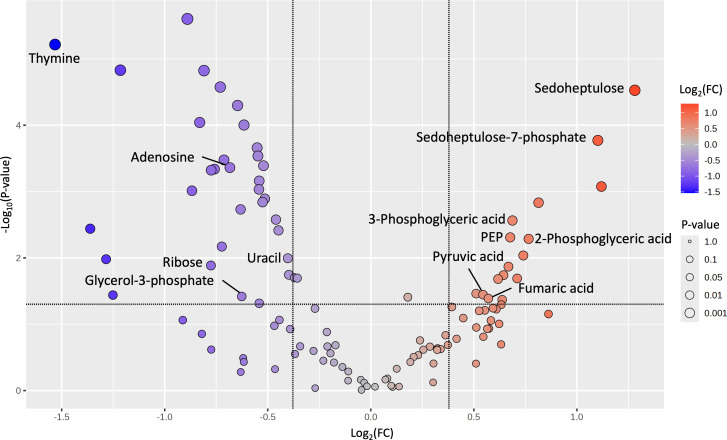
Volcano plot showing intracellular metabolites with significantly higher or lower abundances in ∆MBOVPG45_0748 compared to the parent strain PG45. Each dot in the volcano plot represents an independent metabolite identified by GC-MS. Blue dots above the horizontal dashed line and to the left of the left vertical dashed line indicate metabolites with significantly lower abundance (fold change [FC] < 0.77, *P* < 0.05) in the mutant, while red dots above the horizontal dashed line and to the right of the right vertical dashed line indicate metabolites with significantly higher abundance (FC > 1.3, *P* < 0.05). The size and color intensity of each dot indicate *P* value and fold change, respectively. Metabolites with significantly different abundances that mapped to the previously constructed metabolic map of *M. bovis* (21) are labeled with compound names. PEP: phosphoenolpyruvic acid.

Thymine had significantly lower abundance in ∆MBOVPG45_0748 and had the lowest fold change between the two strains (0.3458 times the relative abundance in the parent strain, *P* < 0.0001), while sedoheptulose had significantly higher abundance and the highest fold change between the two strains (2.4302 times the relative abundance in the parent strain, *P* < 0.0001) (Fig. 2). Significant metabolites were grouped into different pathways based on the recently constructed metabolic map of *M. bovis* (21). Four metabolites in the purine and pyrimidine metabolic pathway (uracil, adenosine, ribose, and thymine) and one metabolite in glycerol metabolism (glycerol-3-phosphate) were detected at significantly lower abundance in ∆MBOVPG45_0748. Amino acids, including cysteine, ornithine, and phosphoserine, as well as sugars, including sucrose, ribose, and mannitol, were also less abundant in ∆MBOVPG45_0748 (Table S1). Additionally, six metabolites involved in the pentose phosphate pathway and glycolysis (sedoheptulose-7-phosphate, 2-phosphoglyceric acid, 3-phosphoglyceric acid, phosphoenolpyruvic acid, pyruvic acid, and fumaric acid) were detected at significantly higher abundance in ∆MBOVPG45_0748 compared to the parent strain (Fig. 2).

### Metabolomic footprinting revealed potential disruption of nutrient uptake in ∆MBOVPG45_0748

**Fig 3:**
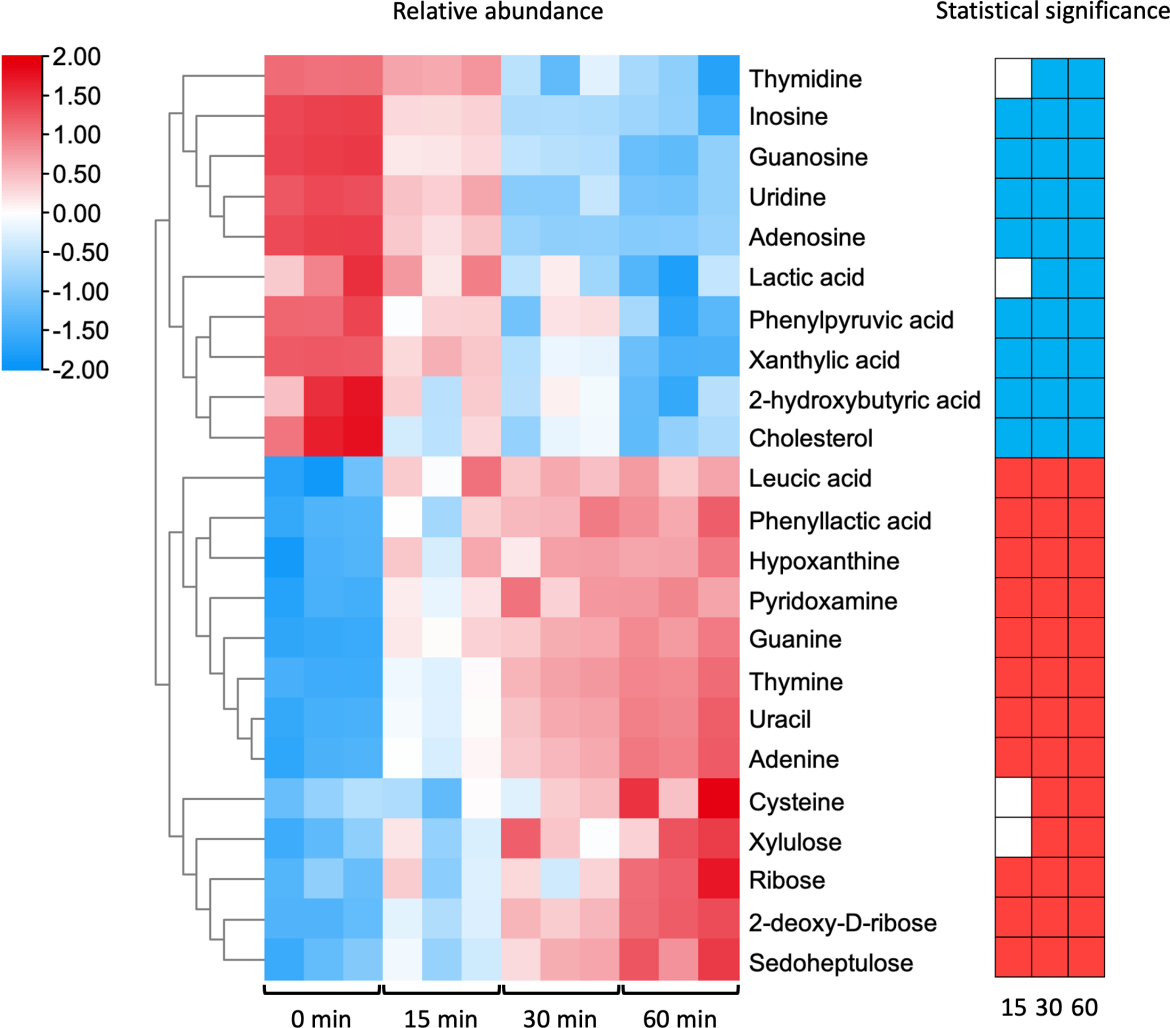
Culture supernatant components with significantly differing abundances in *M. bovis* PG45 in metabolomic footprinting analysis. The left heatmap shows the relative abundance of supernatant components in each of the three replicates after incubation for 0, 15, 30, or 60 min. The right heatmap shows the significance of the change in abundance of each component at the three different timepoints (15/30/60 min) compared with 0 min, with blue indicating significantly lower abundance, red indicating significantly higher abundance, and white indicating no significant change.

Ten components were found to have significantly reduced abundances in the culture supernatant over time in the metabolomic footprinting analysis of the parent strain PG45. There were significantly lower abundances of four nucleosides (adenosine, inosine, guanosine, and uridine), one sterol (cholesterol), and three organic acids (phenylpyruvic acid, xanthylic acid, and 2-hydroxybutyric acid) in the PG45 culture supernatant after incubation for 15 min, 30 min and 60 min (Fig. 3), while thymidine and lactic acid were also found to have lower abundances after incubation for 30 min and 60 min (Fig. 3). Thirteen metabolites had significantly higher abundances in the PG45 culture supernatant over time. Three sugars (ribose, deoxyribose, and sedoheptulose), five nucleobases (guanine, thymine, uracil, hypoxanthine, and adenine), two organic acids (leucic acid and phenyllactic acid), and pyridoxamine were found to have higher abundances at all three timepoints, while xylulose and cysteine had significantly higher abundances after incubation for 30 and 60 min (Fig. 3).

**Fig 4:**
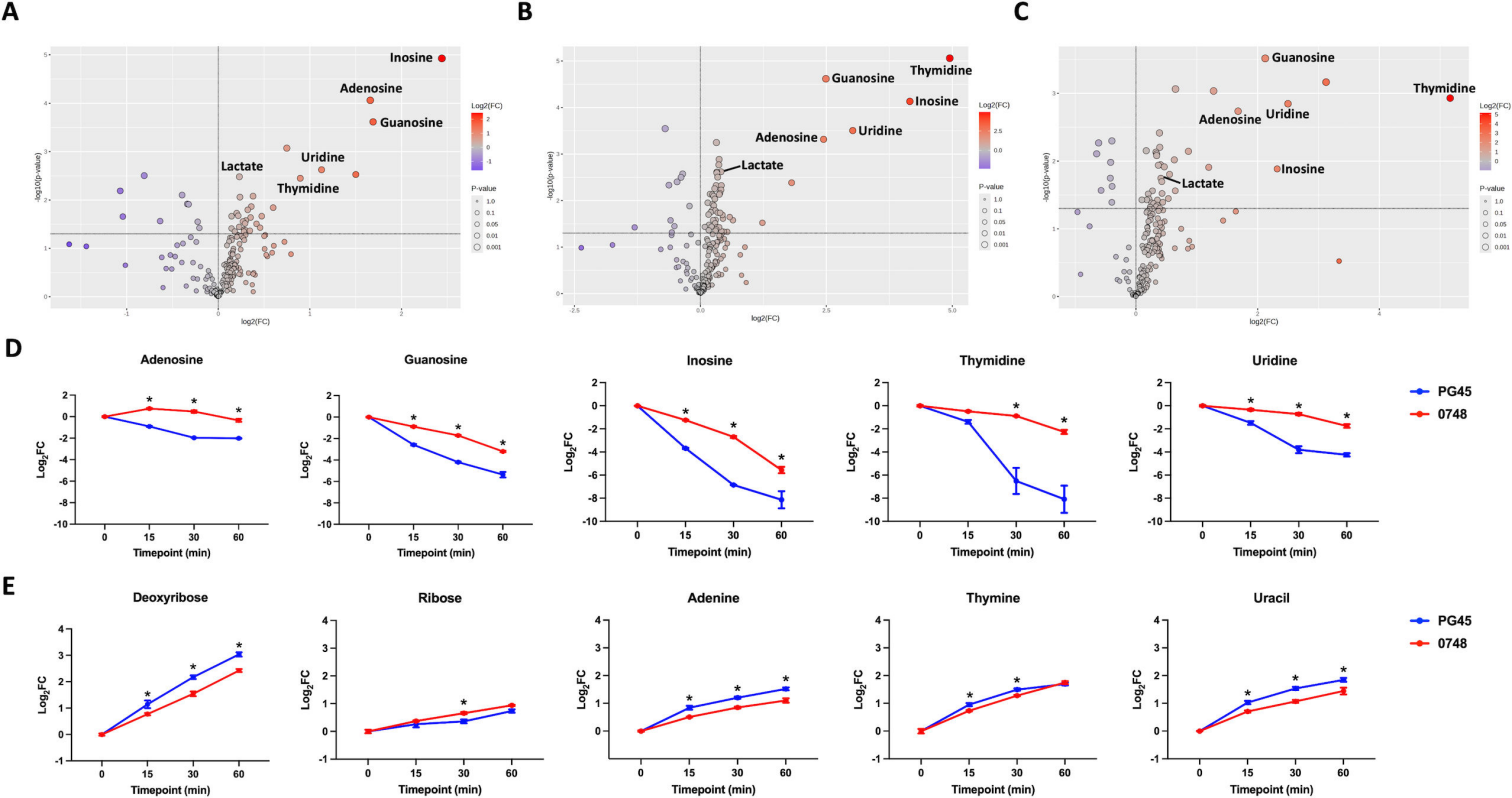
Comparison of metabolomic footprinting profiles of *M. bovis* PG45 and ∆MBOVPG45_0748. Volcano plots showing the components with significantly higher and lower fold changes in the culture supernatants of ∆MBOVPG45_0748 compared with those in the culture supernatants of the parent strain PG45 at (**A**) 15 min, (**B**) 30 min, and (**C**) 60 min. Purple dots above the horizontal dashed line indicate components with significantly lower fold changes (*P* < 0.05) in the culture supernatants of the mutant, while red dots above the horizontal dashed line indicate components with significantly higher fold changes in the culture supernatants of the mutant (*P* < 0.05). The size and color intensity of each dot indicate *P* value and fold change in the statistical analyses, respectively. Log_2_FC values for (**D**) nucleosides and (**E**) their breakdown products at different timepoints. Significant differences in log_2_FC values between ∆MBOVPG45_0748 and PG45 at the same timepoint (*P* < 0.05) are indicated by an asterisk (*).

After incubation for 15 min, the supernatant component with the most significant difference in the rate of depletion between the mutant and the parent was inosine (*P* < 0.0001), with a 5.43 times slower rate of depletion in the culture supernatant of ∆MBOVPG45_0748 than in that of PG45 (Fig. 4A). This was followed by guanosine, with a 3.23 times slower rate of depletion, and adenosine, with a 3.16 times slower rate of depletion, in the supernatant of the ∆MBOVPG45_0748 culture compared to that of the PG45 culture (Fig. 4A). After incubation for 30 min, thymidine was the supernatant component with the most significantly different rate of depletion between the mutant and the parent, with a 30.89 times slower rate of depletion in the culture supernatant of ∆MBOVPG45_0748 (*P* < 0.0001) (Fig. 4B). Its rate of depletion was also 35.88 times slower in the culture supernatant of ∆MBOVPG45_0748 than in that of PG45 at 60 min (*P* < 0.001) (Fig. 4C). In total, five nucleosides had significantly (*P* < 0.05) slower rates of depletion in the culture supernatant of ∆MBOVPG45_0748 (Fig. 4D), while the accumulation rates of deoxyribose at 15, 30, and 60 min were slower in the culture supernatant of ∆MBOVPG45_0748 (*P* value < 0.05), and those of adenine and uracil were also slower at 15, 30, and 60 min (Fig. 4E). Thymine also had a significantly slower rate of accumulation in the culture supernatant of ∆MBOVPG45_0748 at 15 and 30 min (Fig. 4E).

### Isotope labeling demonstrated incorporation of ^13^C_5_-thymidine was disrupted in ∆MBOVPG45_0748

**Fig 5:**
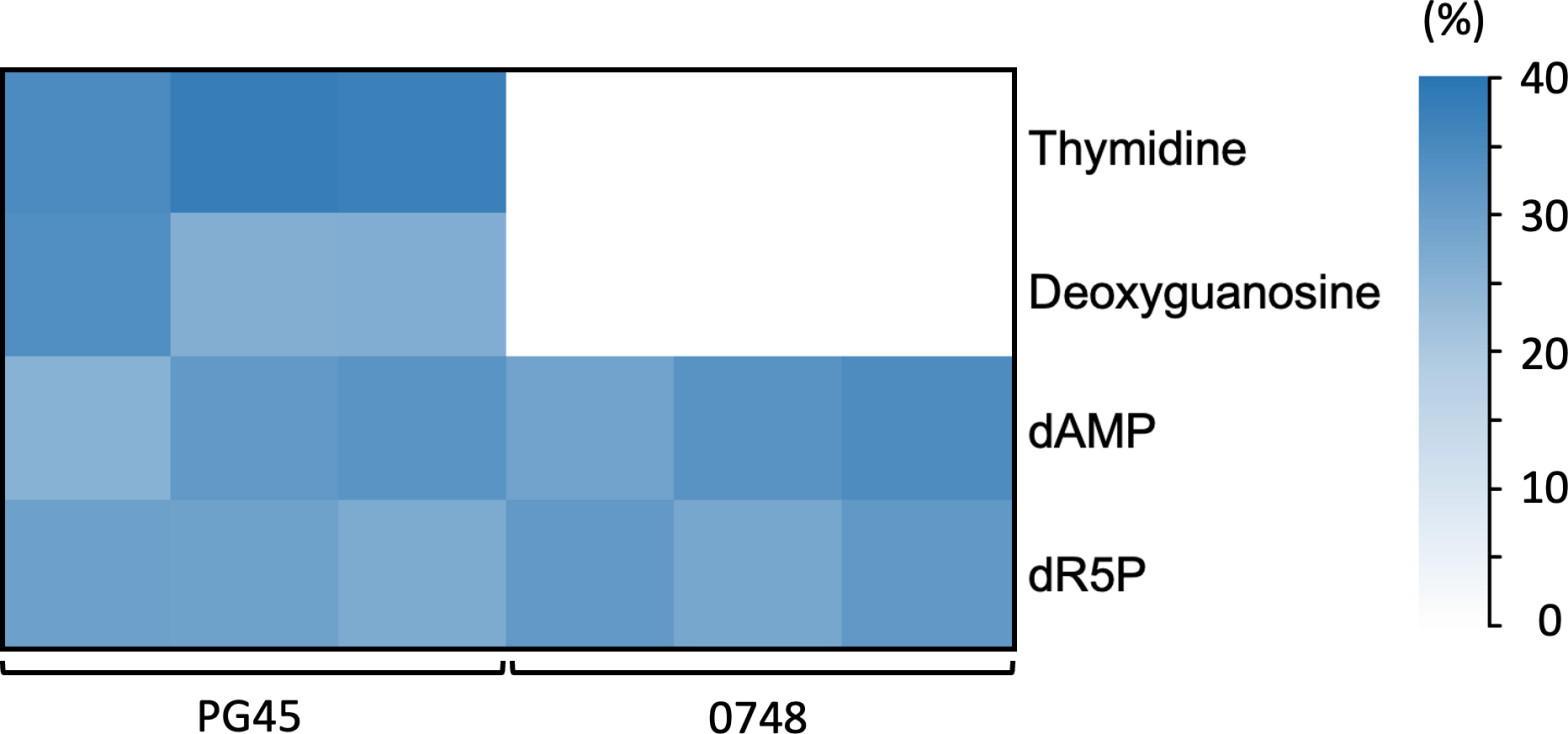
Proportions of M + 5 isotopologues in the total pool of metabolites identified in *M. bovis* PG45 (PG45) and ∆MBOVPG45_0748 (0748). dAMP, deoxyadenosine monophosphate; dR5P, deoxyribose-5-phosphate.

Nucleoside uptake was further studied by culturing the two strains in *M. bovis* growth medium containing ^13^C_5_-thymidine until the mid-logarithmic phase and detecting labeled metabolites by LC-MS (Table S2). Four labeled metabolites were identified in PG45, including two nucleosides (thymidine and deoxyguanosine), one nucleotide, deoxyadenosine monophosphate (dAMP), and one intermediate in nucleoside metabolism, deoxyribose-5-phosphate (dR5P) (Fig. 5). No labeled thymidine or deoxyguanosine was detected in ∆MBOVPG45_0748, while labeled dAMP and dR5P were still identified (Fig. 5).

### Disruption of MBOVPG45_0748 affected the ability of *M. bovis* to utilize extracellular DNA

**Fig 6:**
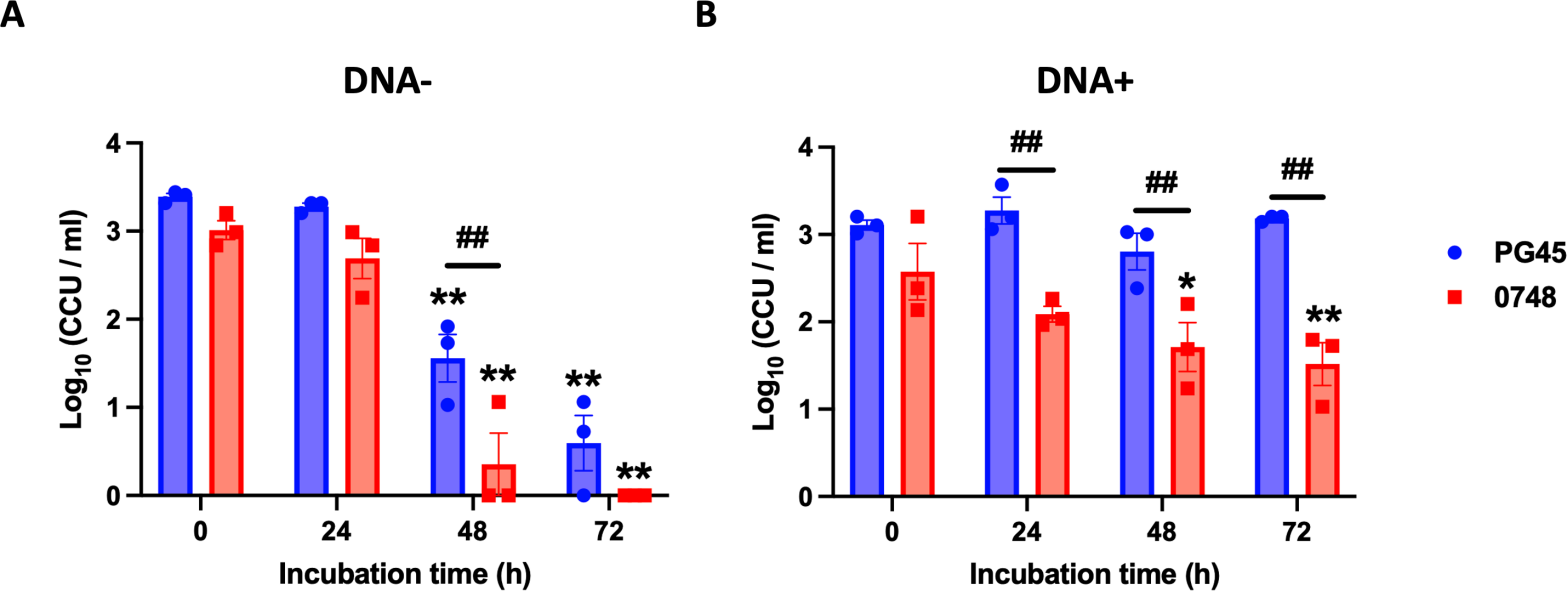
Viable concentrations of *M. bovis* PG45 and ∆MBOVPG45_0748 after incubation in cell culture maintenance medium (**A**) without or (**B**) with additional DNA. *P < 0.05 in the multiple comparisons between 0 h and each of the three later timepoints for the same strain; **P < 0.01 in the multiple comparisons between 0 h and each of the three later timepoints for the same strain; ^##^*P* < 0.01 in the multiple comparisons of PG45 and ∆MBOVPG45_0748 at the same timepoint.

PG45 and ∆MBOVPG45_0748 were cultured in cell culture maintenance medium (DMEM) with or without DNA supplementation to investigate the impact of the disruption of MBOVPG45_0748 on the ability of *M. bovis* to utilize extracellular DNA. Mycoplasma viable cell counts were determined at 0, 24, 48, and 72 h of incubation. A significant decrease in the concentration of viable cells was observed for both strains cultured in cell culture maintenance medium without DNA at 48 and 72 h of incubation (Fig. 6A). There was no significant difference in the viable cell concentrations of the parent strain PG45 and the mutant ∆MBOVPG45_0748 when they were cultured in cell culture maintenance medium without DNA, except at 48 h, when the concentration of viable cells of ∆MBOVPG45_0748 was lower (Fig. 6A).

The concentrations of viable *M. bovis* PG45 when cultured in cell culture maintenance medium supplemented with DNA did not change significantly over the entire incubation period. However, there was a significant decrease in the concentration of viable ∆MBOVPG45_0748 cells after 48 and 72 h of incubation (Fig. 6B). No significant difference was detected between the strains in viable cell concentrations at 0 h, but significantly lower concentrations of viable ∆MBOVPG45_0748 were detected after incubation for 24, 48, or 72 h, compared to PG45 (Fig. 6B).

## DISCUSSION

A combination of intracellular and extracellular metabolomic analyses was used in this study to identify the target nutrients of a transporter operon that has been shown to play a critical role in the survival of *M. bovis* when cultured in association with host cells. This demonstrated the role of this transporter operon in the uptake of nucleosides, as well as in the utilization of extracellular DNA, by *M. bovis*. Metabolomic profiling also revealed potential metabolic adaptations of *M. bovis* during disrupted nucleoside uptake, helping improve our understanding of compensatory strategies used by *M. bovis* in nutrient-deficient conditions.

Mycoplasmas are unable to synthesize purines or pyrimidines and instead import nucleic acid precursors, including nucleosides and nucleobases (22, 23). Amino acids, glycerol, sugars, and organic acids are also important nutrients for mycoplasmas because of their important roles in biopolymer synthesis (24), carbon metabolism (9), and energy generation (25, 26). As MBOVPG45_0749, the gene adjacent to MBOVPG45_0748 in a putative glycerol transporter operon, was not found to be responsible for glycerol uptake in a previous study (16), we first aimed to determine the potential target nutrients of this transporter operon by comparative metabolomic profiling of the ∆MBOVPG45_0748 mutant and its parent strain, PG45. The number of metabolites involved in central carbon metabolism, purine and pyrimidine metabolism, glycerol metabolism, and amino acid metabolism detected in this study (117 metabolites) was greater than the number detected in the initial study used to establish a metabolic map of *M. bovis*, in which 84 metabolites were identified (21). Consistent with the prediction of a conserved glycerol-3-phosphate transporter domain in MBOVPG45_0748, the significantly lower abundance of glycerol-3-phosphate in ∆MBOVPG45_0748 compared to the parent strain suggested that this operon might be involved in the uptake of glycerol-3-phosphate rather than glycerol. Additionally, the absence of MBOVPG45_0748 was also associated with reduced levels of amino acids, sugars, and purine and pyrimidine metabolites, suggesting a possible role for this protein in the uptake of these components.

Previous metabolomic footprinting analyses on *M. bovis* detected significant changes in components in the culture supernatant within 60 min of incubation (21). Therefore, we used this time frame in this study to compare changes in the component profiles in the culture supernatants of the mutant and its parent. *M. bovis* has previously been shown to have a potent membrane nuclease (27) and a nucleoside ABC transport system (11), which indicated that it degraded extracellular nucleic acids to release nucleosides for uptake. This was consistent with the significant depletion of nucleosides from the culture supernatant of *M. bovis* PG45 in the study we report here, suggesting the incorporation of these nutrients by the organism. In a previous study of ruminant mycoplasma species, *M. bovis* was defined as a non-fermenting, non-arginine hydrolyzing mycoplasma species that is unable to oxidize glucose or glycerol and, instead, oxidizes organic acids, including lactic acid, 2-oxobutyrate, and pyruvate (26). The presence of an active gluconeogenic flux from pyruvate to glyceraldehyde-3-phosphate in *M. agalactiae*, supported by *in vitro* assays and proteomics, suggests that pyruvate and lactate might also serve as carbon sources for some mycoplasmas (28). In the study described here, lactic acid and two other organic acids were also depleted in the PG45 strain culture supernatant, suggesting the utilization of these components for energy generation or carbon metabolism. Moreover, the significant depletion of cholesterol in the PG45 culture supernatant detected in our study was consistent with the uptake of this compound by multiple mycoplasma species (29), confirming that cholesterol is an important nutrient for *M. bovis*. In addition to the depletion of nucleosides, our metabolomic footprinting study detected the accumulation of breakdown products of nucleosides, including ribose, deoxyribose, and nucleobases, in the culture supernatant. This suggests that nucleosides might be further degraded by *M. bovis* to release these products.

**Fig 7:**
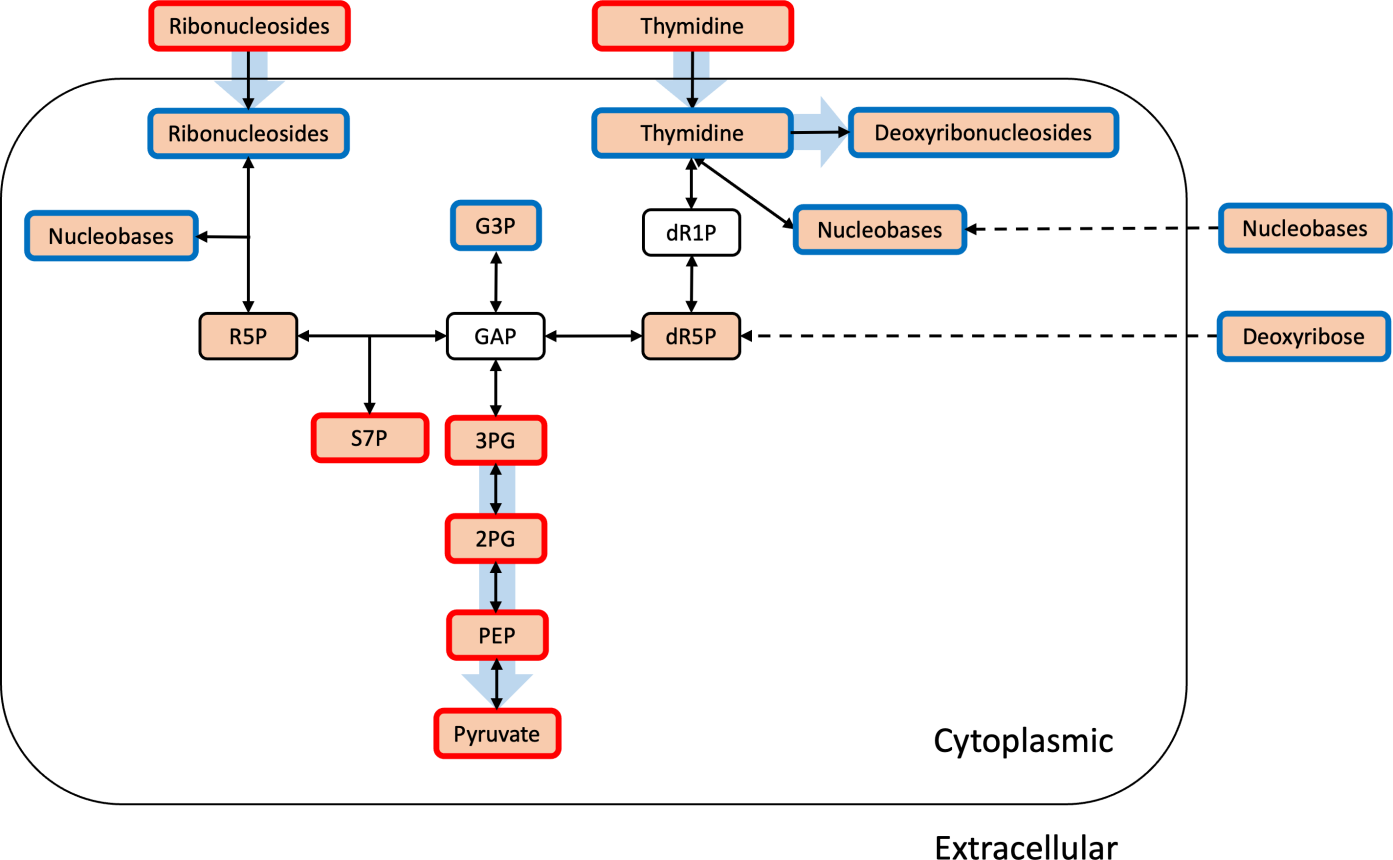
Schematic overview of the metabolic pathways affected by the mutation in ∆MBOVPG45_0748, compared to the parent strain PG45. Orange rectangles indicate metabolites that were detectable using metabolomic analyses, and white rectangles indicate metabolites that were not detectable. Red outlines indicate intracellular metabolites with a higher intracellular abundance in ∆MBOVPG45_0748 compared to PG45 or extracellular supernatant components with slower rates of depletion in ∆MBOVPG45_0748 compared to PG45. Blue outlines indicate metabolites with a lower intracellular abundance in ∆MBOVPG45_0748 compared to PG45, or extracellular supernatant components with slower rates of accumulation in ∆MBOVPG45_0748 compared to PG45. Black outlines indicate metabolites with no difference in abundance between ∆MBOVPG45_0748 and PG45. Black arrows indicate metabolic pathways identified previously (21) and in our study, while dashed arrows indicate metabolic pathways inferred from our study. Blue arrows indicate metabolic fluxes downregulated in ∆MBOVPG45_0748 compared with PG45, as inferred from this study. dR1P, deoxyribose-1-phosphate; dR5P, deoxyribose-5-phosphate; GAP, glyceraldehyde-3-phosphate; G3P, glycerol-3-phosphate; PEP, phosphoenolpyruvic acid; R5P, ribose-5-phosphate; S7P, sedoheptulose-7-phosphate; 3PG, 3-phosphoglyceric acid; 2PG, 2-phosphoglyceric acid.

Comparisons of the rates of change in the levels of glycerol-3-phosphate in the culture supernatants detected no difference between ∆MBOVPG45_0748 and its parent strain, PG45, suggesting that the transporter operon was not involved in the incorporation of glycerol-3-phosphate. Instead, the significantly lower rates of depletion of inosine and thymidine from the culture supernatant of ∆MBOVPG45_0748 suggested that the transporter operon had a role in nucleoside uptake. Additionally, the lower rates of accumulation of deoxyribose and nucleobases in the culture supernatant of ∆MBOVPG45_0748 suggested that, in the absence of the transport system encoded by the operon that includes the MBOVPG45_0748 gene, the mutant may have an alternative strategy for maintaining intracellular nucleobases and recycling deoxyribose, thereby compensating for its reduced uptake of inosine and thymidine (Fig. 7). Previous metabolomic studies of *M. bovis* have proposed a connection between glycerol metabolism and nucleotide biosynthesis via the pentose phosphate pathway or, putatively, via deoxyribose-5-phosphate aldolase (21). In our study, the higher abundance of sedoheptulose-7-phosphate in the mutant further suggested that the lower abundance of glycerol-3-phosphate might result from an enhanced utilization of glycerol metabolites for purine and pyrimidine metabolism through the pentose phosphate pathway.

The absence of labeled thymidine and deoxyguanosine in the cells of the ∆MBOVPG45_0748 mutant in our stable isotope tracing study confirmed that the mutation had disrupted nucleoside uptake and interconversion, as suggested by both the steady-state and metabolomic footprinting analyses. Interestingly, the nucleotide dAMP and a nucleoside metabolite dR5P were still found to be labeled in ∆MBOVPG45_0748. Our metabolomic footprinting study suggested that nucleosides were degraded by *M. bovis* to release deoxyribose and nucleobases, and the lower rates of accumulation of these components in the culture supernatant of ∆MBOVPG45_0748 indicated that the uptake of this component for intracellular recycling was upregulated in the mutant. Some bacterial species have been shown to possess deoxyribokinase activity, which enables the phosphorylation of deoxyribose to dR5P (30). The shunt of ^13^C into dR5P and the nucleotide dAMP in ∆MBOVPG45_0748 suggested the extracellular release of labeled deoxyribose from ^13^C_5_-thymidine and subsequent uptake and utilization of this component in downstream pathways, which may act as a compensatory strategy for the maintenance of nucleic acid biosynthesis in the mutant. Further experiments are needed to investigate any upregulation of other nutrient transport systems in this mutant.

In addition to the utilization of carbon from glycolytic intermediates for nucleoside biosynthesis, an active metabolic pathway for the shunt of carbon from nucleosides into glycolysis has also been suggested by the identification of thymidine as a carbon source for *M. hominis* and ribose as a carbon source for *M. pneumoniae* (31, 32). As dR5P is an important intermediate in the catalytic pathway of deoxyribonucleosides (33), the labeling of dR5P detected in our study suggested deoxyribonucleosides could be a carbon source for *M. bovis*. This was also supported by an increased abundance of intermediates of glycolysis and the disrupted nucleoside uptake in ∆MBOVPG45_0748. In microorganisms, disruption of carbon utilization has been shown to affect metabolite abundance in central carbon metabolism. Removal of a carbon source in the culture medium of yeast reduces the metabolic flux in glycolysis, resulting in the accumulation of lower glycolytic intermediates (34). Similar changes have also been seen in *Mycoplasma gallisepticum*, in which the disruption of the incorporation of glycerol, an important carbon source for the organism, significantly increased the abundance of glycolytic intermediates, including 3-phosphoglyceric acid and 2-phosphoglyceric acid (9). In our study, an increased abundance of intermediates of glycolysis in the cytoplasm of the ∆MBOVPG45_0748 mutant suggested a reduced metabolic flux in glycolysis and thus accumulation of glycolysis metabolites (Fig. 7).

The MnuA extracellular nuclease (27) and the 5′-nucleotidase (35) of *M. bovis* could contribute to degradation of extracellular DNA into nucleotides and nucleosides for subsequent uptake. Given the function of MBOVPG45_0748 in the transport of nucleosides revealed by our metabolomic analysis, we further aimed to clarify the role of MBOVPG45_0748 in the utilization of extracellular DNA by comparing the capacity of the mutant and its parent to survive in the nutrient-limited conditions of cell culture maintenance medium with or without additional DNA. It has been shown previously that DMEM-based cell culture maintenance medium is not able to sustain growth of *M. bovis*, but supplementation with DNA significantly enhances its proliferation in both axenic culture and in association with host cell cultures (36). In our present study, supplementation of the cell culture maintenance medium with DNA improved the survival of both the parent strain and ∆MBOVPG45_0748, with a more pronounced effect on the parental PG45 strain. These results further confirmed the role of the MBOVPG45_0748 gene in the utilization of extracellular DNA, consistent with the requirement of this gene for efficient importation of nucleosides. The beneficial effect of extracellular DNA on the survival of ΔMBOVPG45_0748 may be due to the presence of alternative nucleoside transport systems, as a previously identified nucleoside ABC transporter system, UptBACD, found in *M. bovis* strain M23 is also conserved in strain PG45 (11). Phosphodiesterases with cyclic dinucleotide and nanoRNA degradation activity have been shown to be essential for proliferation of *M. bovis* in association with host cells (37). RNA sequencing in a previous study also showed that the phosphodiesterase activity could alter the expression of immune response-associated genes in host cells infected with *M. bovis*, suggesting the regulatory role of this activity in host cellular processes (38). These studies and our findings suggest that efficient purine and pyrimidine uptake and recycling is a critical contributor to the interactions between *M. bovis* and host cells.

In conclusion, our integrated metabolomic profiling studies have identified a novel nucleoside transporter in *M. bovis* that plays an important role in purine and pyrimidine metabolism. The insertion of the transposon could have had polar effects on the expression of the MBOVPG45_0747 gene, which is located downstream of MBOVPG45_0748 in the same ABC transporter operon. However, the impact of this would not be expected to have been significantly different from disruption of the expression of the MBOVPG45_0747 gene, as the products of both genes are predicted to be components of the same transporter system. Our studies also highlighted the dependence of *M. bovis* on efficient purine and pyrimidine metabolism for survival in association with host cells.

## Data Availability

Raw data matrices generated by GC-MS and LC-MS in this study are provided as supplemental material (Tables S2–S4).

## SUPPLEMENTAL MATERIAL

Figures S1 to S3 aem.01298-25-s0001.docx Fig. S1: Growth curves of *M. bovis* PG45 and ∆MBOVPG45_0748 (0748) in *M. bovis* growth medium. Fig. S2: Predicted transmembrane structure of MBOVPG45_0748, with six transmembrane helices predicted by DeepTMHMM. Fig. S3: Principal component analysis (PCA) of the metabolic profiles of *M. bovis* PG45 (PG45) and ∆MBOVPG45_0748 (0748).

Table S1 aem.01298-25-s0002.xlsx Significantly different metabolites in ∆MBOVPG45_0748 compared with *M. bovis* PG45.

Table S2 aem.01298-25-s0003.xlsx Peak areas of isotopologues in the stable isotope labeling study.

Table S3 aem.01298-25-s0004.xlsx Raw data matrix generated by GC-MS in steady-state metabolomic analysis.

Table S4 aem.01298-25-s0005.xlsx Raw data matrix generated by GC-MS in metabolomic footprinting analysis.

ASM does not own the copyrights to Supplemental Material that may be linked to, or accessed through, an article. The authors have granted ASM a non-exclusive, world-wide license to publish the Supplemental Material files. Please contact the corresponding author directly for reuse.
