# Supplementary material for: Dependence of Mycoplasma bovis on a novel nucleoside transporter for survival in association with host cells
Source: Appl Environ Microbiol. 2025 Dec 12;92(1):e01298-25. doi: 10.1128/aem.01298-25 (PMC12838404; doi:10.1128/aem.01298-25)
Supplement: Figures S1 to S3 — Fig. S1: Growth curves of M. bovis PG45 and ∆MBOVPG45_0748 (0748) in M. bovis growth medium. Fig. S2: Predicted transmembrane structure of MBOVPG45_0748, with six transmembrane helices predicted by DeepTMHMM. Fig. S3: Principal component analysis (PCA) of the metabolic profiles of M. bovis PG45 (PG45) and ∆MBOVPG45_0748 (0748). [file aem.01298-25-s0001.docx]

**Supplementary figures**

**
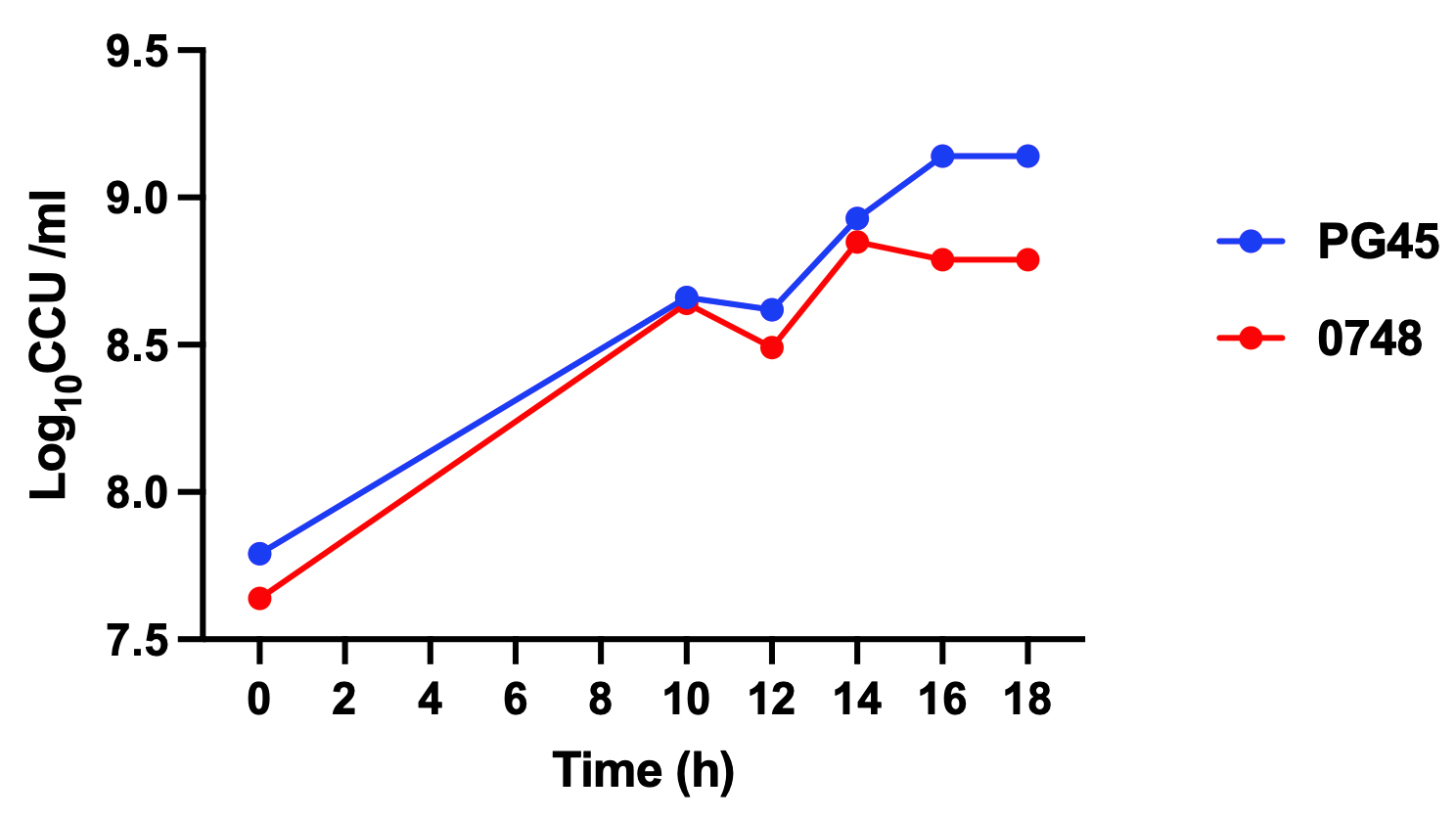
**

**Figure S1.** Growth curves of *M. bovis* PG45 and ∆MBOVPG45_0748 (0748) in *M. bovis* growth medium.

**
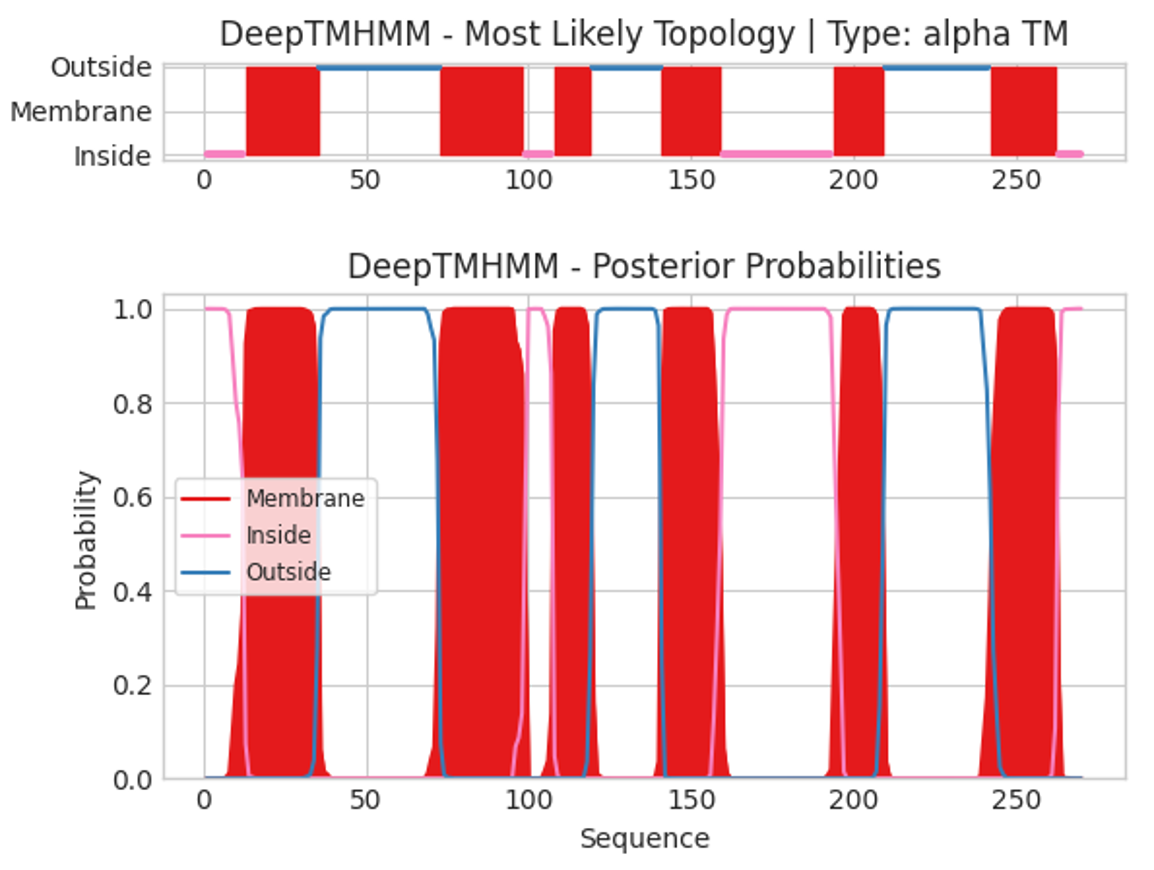
**

**Figure S2.** Predicted transmembrane structure of MBOVPG45_0748, with six transmembrane helixes predicted by DeepTMHMM.


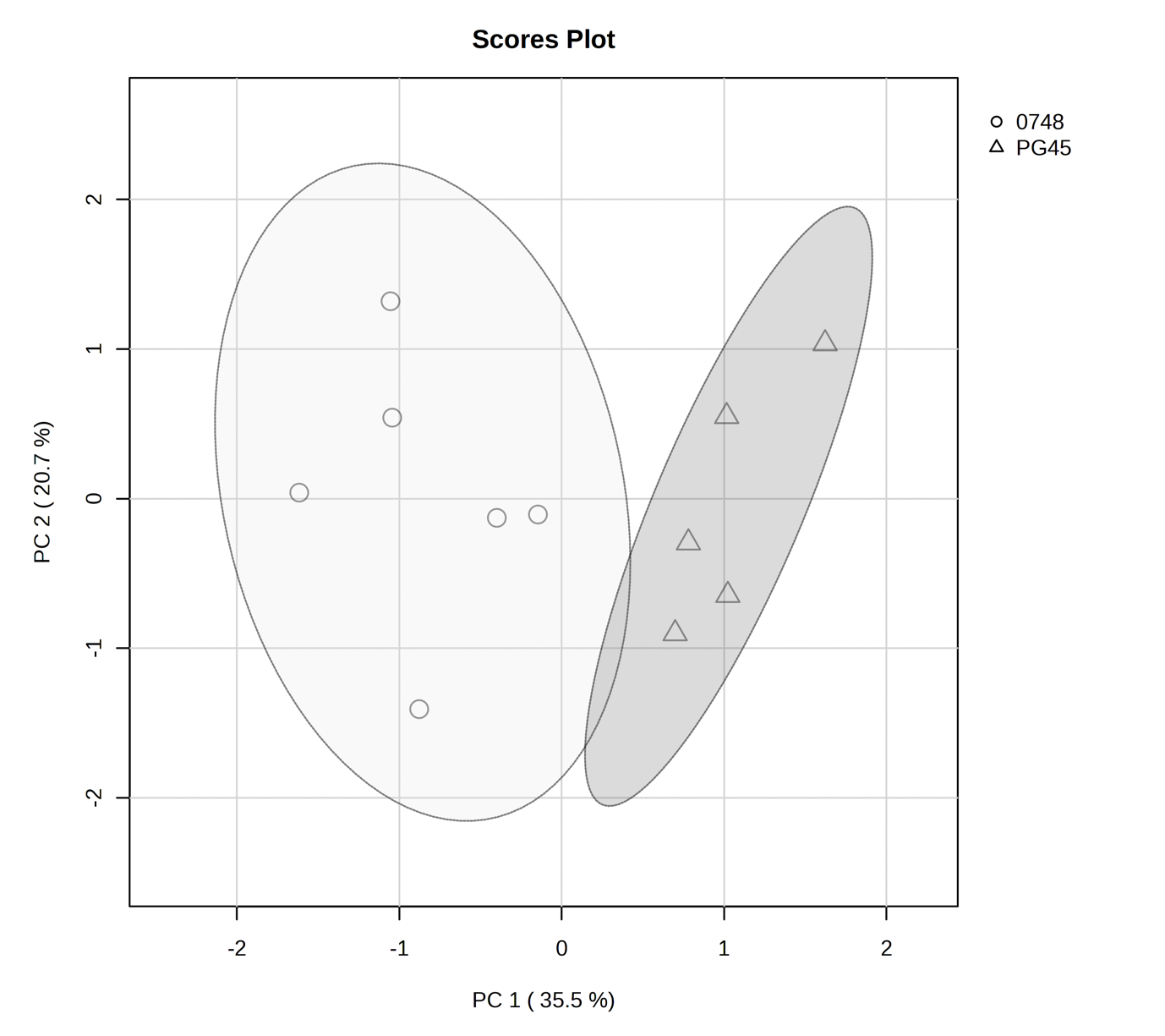


**Figure S3.** Principal component analysis (PCA) of the metabolic profiles of *M. bovis* PG45 (PG45) and ∆MBOVPG45_0748 (0748), with the 95% confidence areas shown as shaded ovals for each strain.
